# Supplementary material for: Dysregulation of lung epithelial cell homeostasis and immunity contributes to Middle East respiratory syndrome coronavirus disease severity
Source: mSphere. 2025 Jan 30;10(2):e00951-24. doi: 10.1128/msphere.00951-24 (PMC11853001; doi:10.1128/msphere.00951-24)
Supplement: Supplemental material — Supplemental figures and tables. [file msphere.00951-24-s0002.pdf]

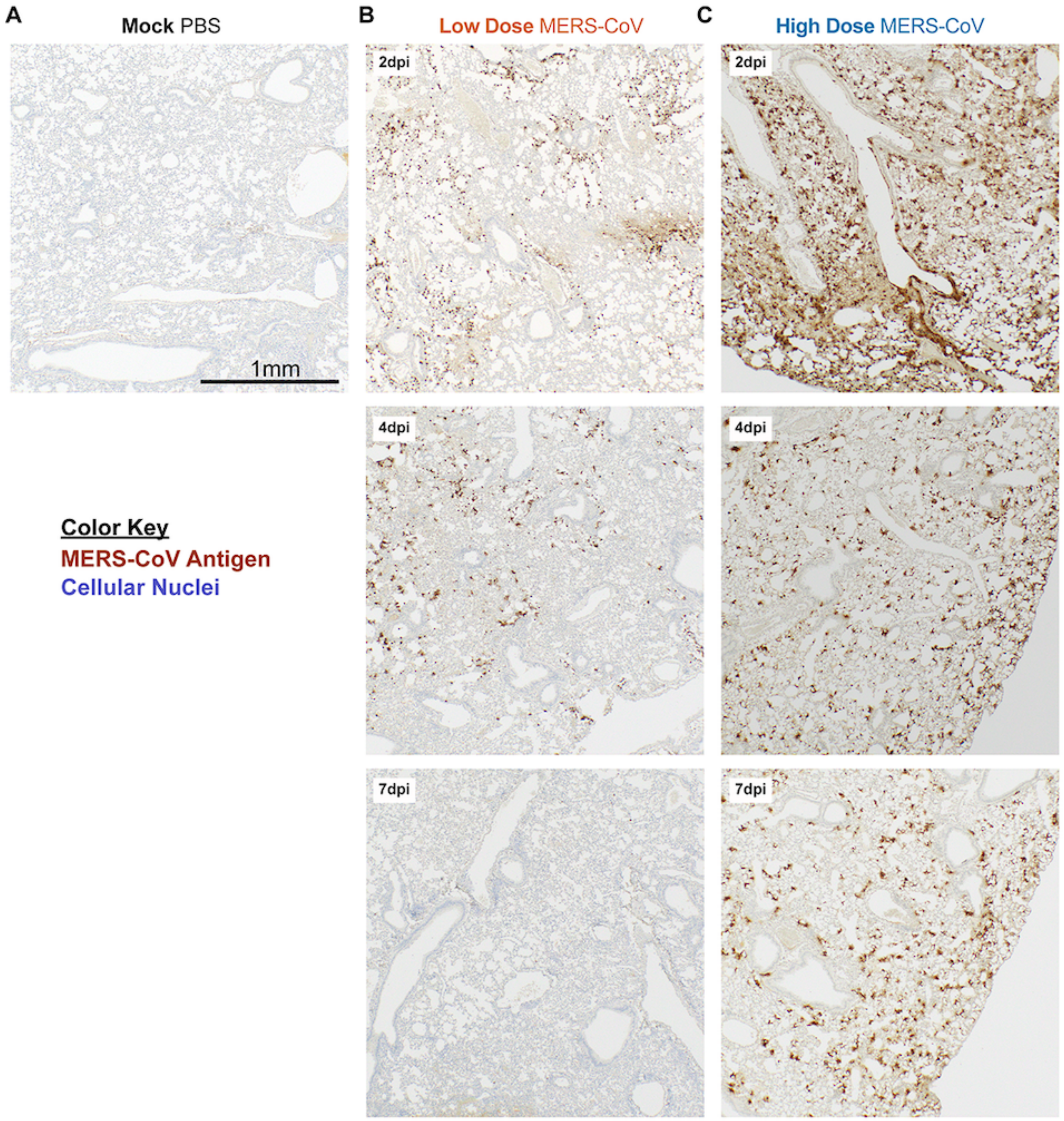

Figure S1: Levels of MERS-CoV viral antigen is dose dependent in lung tissue sections. Related to Main Figure 1. Lung tissue sections from the study described in Main Figure 1 were labeled for MERS-CoV antigen (brown) and counterstained with hematoxylin (blue). Representative images from 2, 4 and 7 dpi are shown.

Scoring scheme for lung discoloration “hemorrhage” score

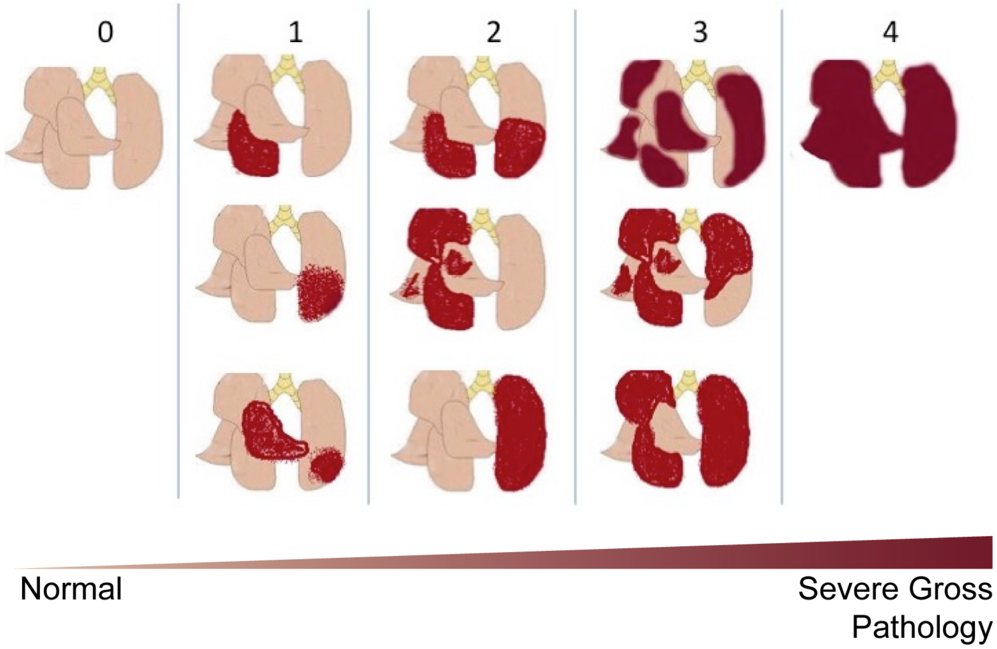

Figure S2: Lung discoloration scoring scheme. Lung discoloration is a gross pathological phenotype associated with emerging coronavirus infection in mice. We have developed a scale for scoring this phenotype where a 1 is a normal and pink healthy lung and a 4 is deep red coloring with a hemorrhage like appearance.

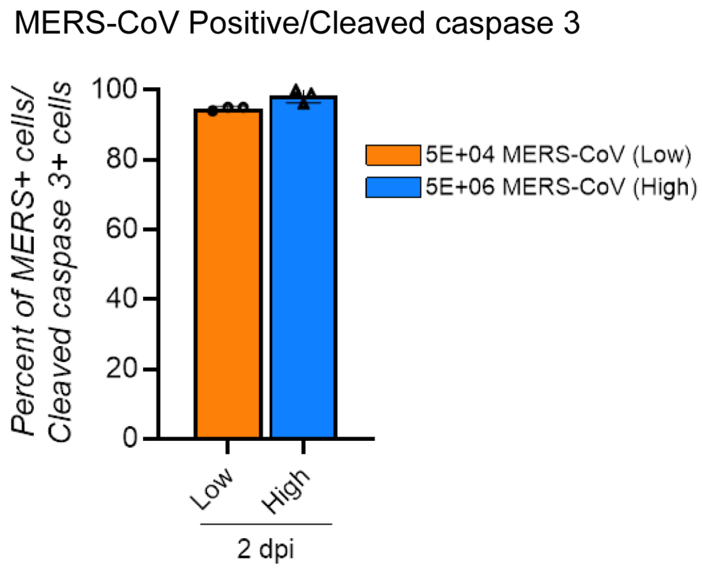

Figure S3: Percentage cleaved caspase 3 and MERS-CoV positive cells. Related to Main Figure 3. One hundred cleaved caspase 3 positive cells from three Low and High Dose MERS-CoV groups from 2 dpi were evaluated for MERS-CoV antigen co-positivity. The percent cleaved caspase 3 and MERS-CoV antigen positive is displayed.

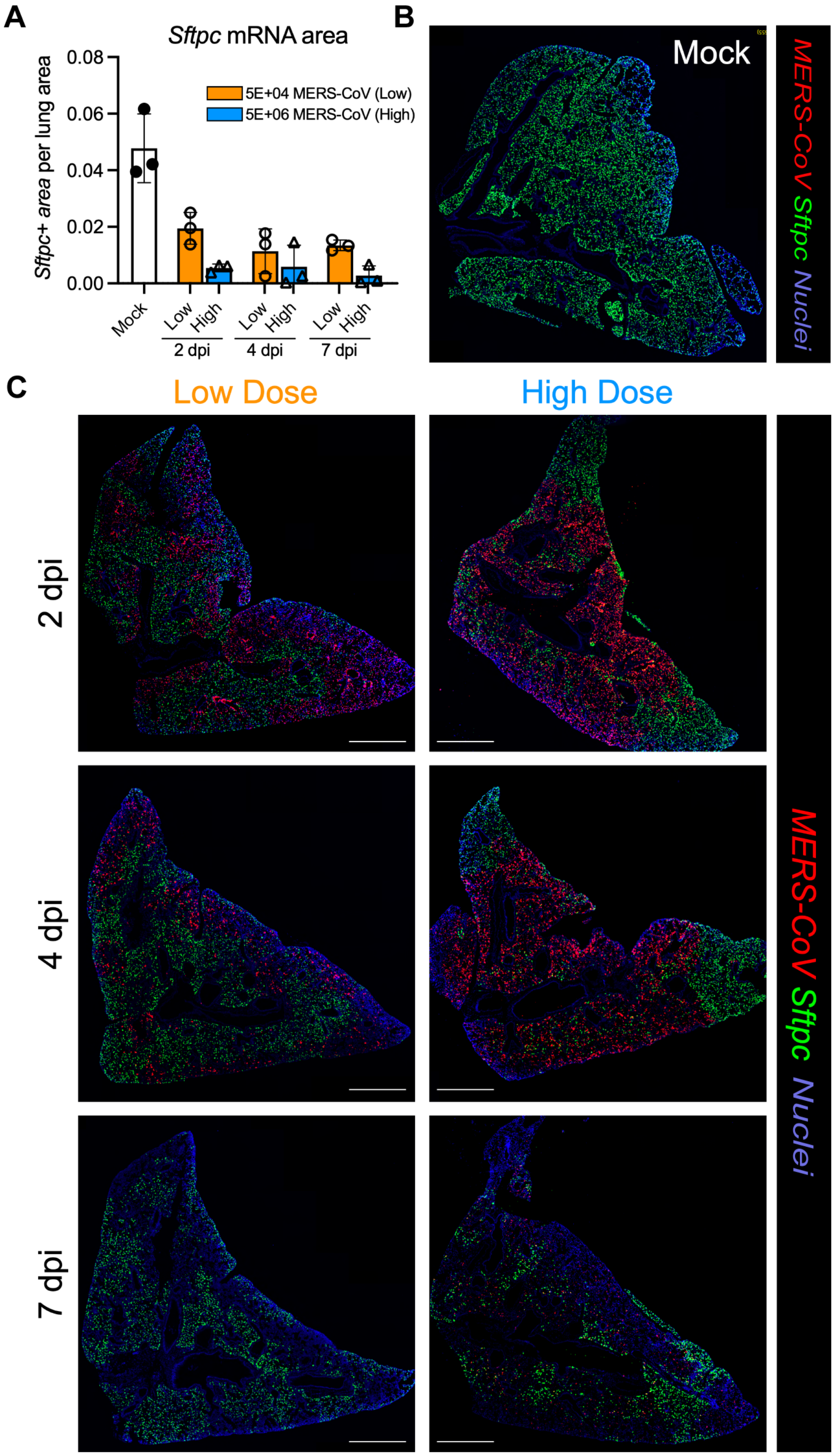

Figure S4: Surfactant expression is diminished with severe MERS-CoV disease and is not recovered by 7dpi. RNAscope in situ hybridization for MERS-CoV and *Sftpc* RNA for mock, Low and High dose infection conditions at 2, 4 and 7dpi. **(A)** Quantitation of *Sftpc* RNA labeled using RNAscope. RNAscope in situ hybridization for MERS-CoV and *Sftpc* RNA for mock (**B**), Low and High dose infection conditions at 2, 4 and 7dpi **(C).**

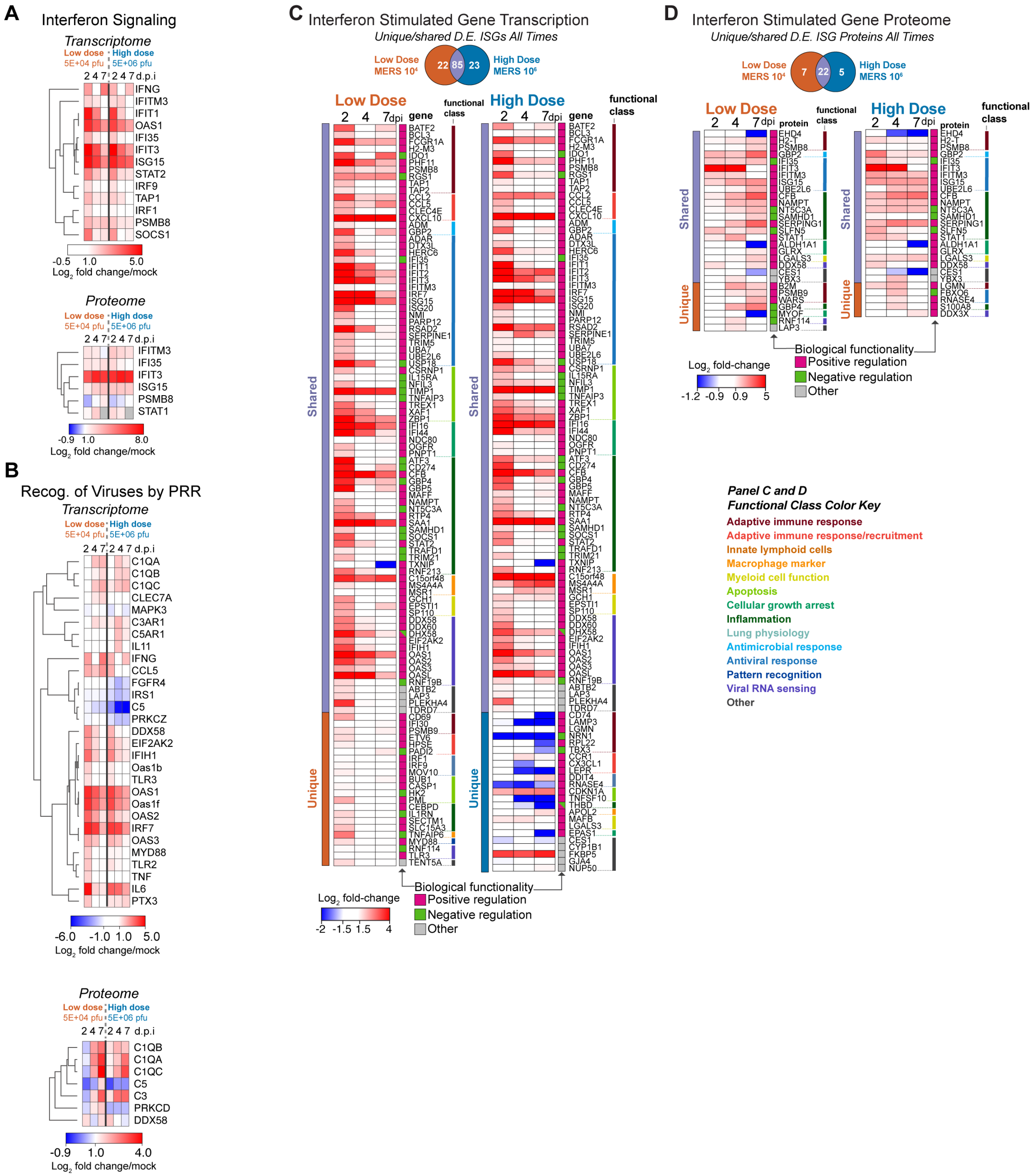

Figure S5: Most innate immune related gene expression is similar across MERS-CoV dose groups. (A-B) Significantly regulated transcripts and proteins for innate immune response related canonical pathways from **"Figure 4"**: **(A)** Interferon Signaling and **(B)** Recognition of Viruses by Pattern Recognition Receptors **(B)**. **(C-D)** Transcriptomic and proteomic data was filtered through a list of 390 known interferon stimulated genes (ISGs) (1). **(C)** Heat map of the differentially regulated ISG transcripts **(C)** or proteins **(D)** ($\log_2$ fold-change) for both high and low dose MERS-CoV infection. Gene ontology information (biological function, positive/negative regulatory role) is also noted.

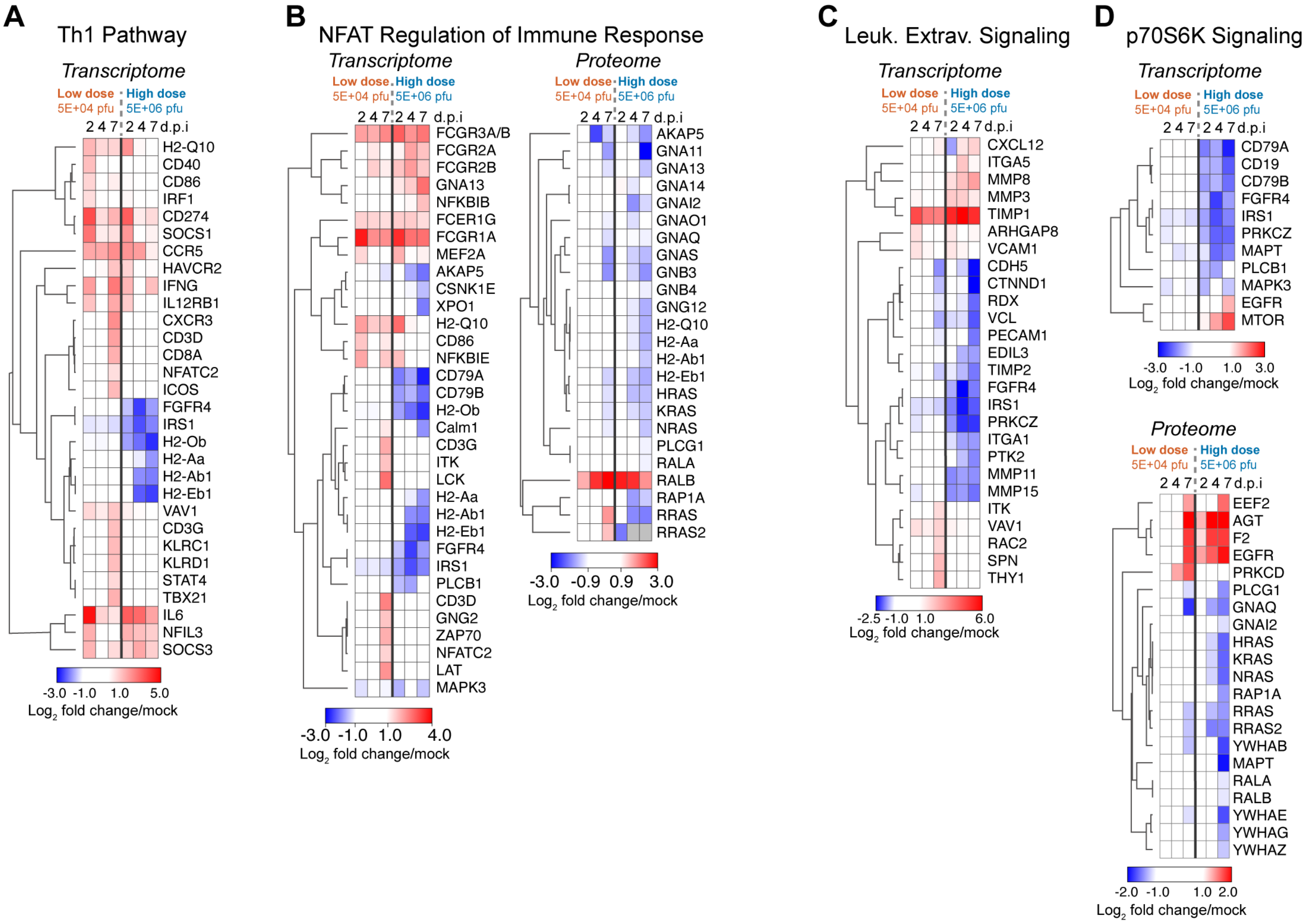

Figure S6: High dose MERS-CoV infection is associated with a downregulation of adaptive immune gene and protein expression. Significantly regulated transcripts and proteins for adaptive immune response related canonical pathways from **"Figure 4":** Th1 Pathway **(A)**, NFAT Regulation of Immune Response **(B)**, Leukocyte Extravasation Signaling **(C)**, and p70S6K Signaling **(D)**. All heat maps show log_2 fold-change over mock data per gene/protein significantly affected for each pathway.

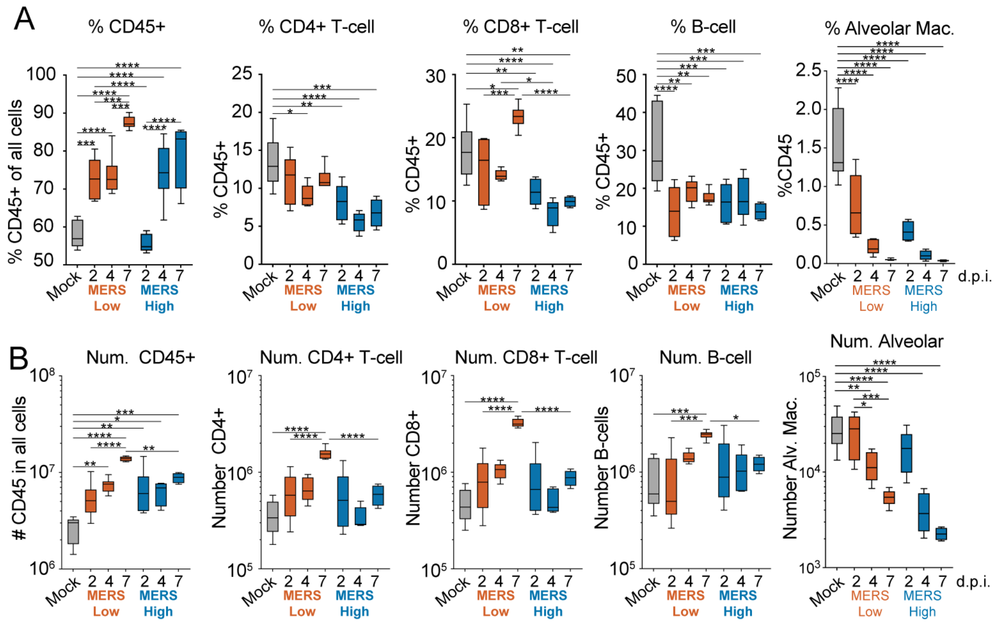

Figure S7: Severe MERS-CoV infection is associated with a failure to recruit adaptative immune cells. Related to Main Figure 5. 22 week old female C57BL/6 hDPP4 were intranasally infected with PBS ("mock", N = 9), $5x10^4$ PFU mouse adapted MERS ma1 ("low dose", N = 22) or $5x10^6$ PFU MERS ma1 ("high dose", N = 24) in a 50μl volume. On 2, 4, and 7 days post-infection, six mice per infected dose group (3 male and 3 female) or 3 mock infected mice were humanely sacrificed, lung tissue was perfused with DPBS, and single cell suspensions of lung leukocytes were counted and stained for analysis by flow cytometry. **(A)** Frequencies of leukocytes (CD45+), T cells (CD3+, CD4+), CD8+ T-cell (CD3+, CD8+), B cells (CD19+) and alveolar macrophage (CD45+, CD11b-, CD103-, SiglecF+, CD11c+). **(B)** The numbers of cells in "A". Asterisks indicate statistically significant differences by one-way ANOVA followed by Sidak's multiple comparison test.

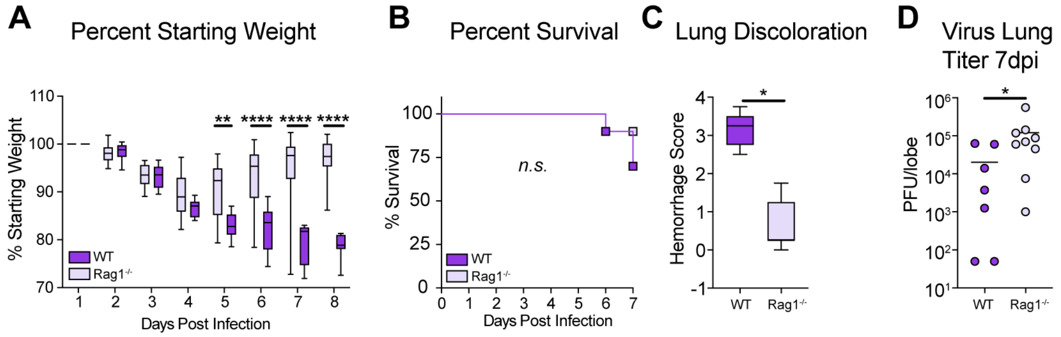

Figure S8: Functional T and/or B-cells contribute to MERS-CoV disease and are important for controlling MERS-CoV replication. Related to Main Figure 6. **(A)** Percent starting weight of 10-week old male and female WT C57BL/6 hDPP4 mice or C57BL/6 hDPP4 RAG1$^{-/-}$ mice infected with 2.4x10^{4} PFU of the further mouse adapted MERS m35C4 strain (WT, N = 11; RAG1$^{-/-}$ N = 10). Asterisks denote statistical significance by two-way ANOVA with a Sidak's multiple comparison test. **(B)** Percent Survival. **(C)** Gross lung pathology "lung discoloration". Asterisks denote statistical significance as determined by Wilcoxon matched-pairs signed rank test. **(D)** Virus lung titer on 7dpi by plaque assay. Asterisks denote statistical significance as determined by Mann Whitney test.

Supplemental Table 1: Genetic changes of mouse adapted MERS-CoV strains compared to MERS-CoV EMC/2012.

Gene/Location	5' UTR	nsp3	nsp6	nsp8	nsp14	Spike	Orf3	Orf4a	orf4b	Orf M
MERS-CoV MA1	(ΔA, nucleotide 2	A217V	T184I	I108L		R884-RMR insertion and S885L			ΔE45-H243 (nt 26,226 to 26,821)	
MERS-CoV m35C4	A:G, nucleotide 28	A217V and T1615N	L232F	I108L	T521I	N222Y, spike R884-RMR insertion and S885L	Q4 stop codon	P85L	orf4b/orf5 deletion (nucleotide 26211-26863)	S2F

References

1. Schoggins JW, Wilson SJ, Panis M, Murphy MY, Jones CT, Bieniasz P, Rice CM. 2011. A diverse range of gene products are effectors of the type I interferon antiviral response. Nature 472:481-5.
